# Asymptomatic *Plasmodium falciparum* malaria prevalence among adolescents and adults in Malawi, 2015–2016

**DOI:** 10.1038/s41598-020-75261-9

**Published:** 2020-10-30

**Authors:** Hillary M. Topazian, Austin Gumbo, Sydney Puerto-Meredith, Ruth Njiko, Alexis Mwanza, Michael Kayange, David Mwalilino, Bernard Mvula, Gerald Tegha, Tisungane Mvalo, Jessie K. Edwards, Michael Emch, Audrey Pettifor, Jennifer S. Smith, Irving Hoffman, Steven R. Meshnick, Jonathan J. Juliano

**Affiliations:** 1 Department of Epidemiology, University of North Carolina, Chapel Hill, NC 27510 USA; 2 National Malaria Control Programme, Malawi Ministry of Health, Lilongwe, Malawi; 3 University of North Carolina Project-Malawi, Lilongwe, Malawi; 4 National HIV Reference Laboratory, Malawi Ministry of Health, Lilongwe, Malawi; 5 Department of Pediatrics, School of Medicine, University of North Carolina, Chapel Hill, NC USA; 6 Department of Geography, University of North Carolina, Chapel Hill, NC USA; 7 Carolina Population Center, University of North Carolina, Chapel Hill, NC USA; 8 Institute for Global Health and Infectious Diseases, University of North Carolina, Chapel Hill, NC USA; 9 Division of Infectious Diseases, School of Medicine, University of North Carolina, Chapel Hill, NC USA

**Keywords:** Malaria, Epidemiology

## Abstract

Malaria remains a significant cause of morbidity and mortality in Malawi, with an estimated 18–19% prevalence of *Plasmodium falciparum* in children 2–10 years in 2015–2016. While children report the highest rates of clinical disease, adults are thought to be an important reservoir to sustained transmission due to persistent asymptomatic infection. The 2015–2016 Malawi Demographic and Health Survey was a nationally representative household survey which collected dried blood spots from 15,125 asymptomatic individuals ages 15–54 between October 2015 and February 2016. We performed quantitative polymerase chain reaction on 7,393 samples, detecting an overall *P. falciparum* prevalence of 31.1% (SE = 1.1). Most infections (55.6%) had parasitemias ≤ 10 parasites/µL. While 66.2% of individuals lived in a household that owned a bed net, only 36.6% reported sleeping under a long-lasting insecticide-treated net (LLIN) the previous night. Protective factors included urbanicity, greater wealth, higher education, and lower environmental temperatures. Living in a household with a bed net (prevalence difference 0.02, 95% CI − 0.02 to 0.05) and sleeping under an LLIN (0.01; − 0.02 to 0.04) were not protective against infection. Our findings demonstrate a higher parasite prevalence in adults than published estimates among children. Understanding the prevalence and distribution of asymptomatic infection is essential for targeted interventions.

## Introduction

Malaria remains a significant cause of morbidity and mortality in Malawi, with an estimated 7.0 million confirmed cases reported in 2018^1^. *Plasmodium falciparum* is the most virulent of the malaria species, giving rise to 98% of infections in-country^1^. While children report the highest proportion of clinical disease^2^, adults are thought to be an important reservoir to sustained transmission due to persistent asymptomatic infection. Underlying infection is unlikely to be diagnosed and treated as individuals often do not exhibit signs and symptoms of disease and consequentially do not seek care. Even if an individual is tested, parasite densities in asymptomatic infections are usually low and may be undetectable by microscopy or rapid diagnostic test (RDT)^3,4^, further reducing the likelihood of diagnosis and treatment. Asymptomatic infections can persist for months and older age is associated with an increased duration of persistent infection, allowing the parasite the opportunity to transmit for a prolonged period of time^5^. The annual *P. falciparum* parasite rate was estimated to be 18–19% in children ages 2–10 years in Malawi in 2015–2016^6^, however, little is known about the prevalence of adult asymptomatic *P. falciparum* infection.

Malaria is geographically and temporally heterogenous. In Malawi, malaria risk has been found to be modified by several environmental factors, including elevation, rainfall, temperature, and proximity to active agriculture^7–9^. Insecticide-treated bed nets (ITN) are Malawi’s primary vector control strategy^10^, with bed nets given to all households with pregnant women, newborn babies, and to the general population every three years through mass distribution campaigns^11^. ITNs act as both a physical and chemical barrier to repel and kill mosquitoes which land on the net^12^. Bed net use has been shown to have protective individual and community-level associations with *P. falciparum* infection in children under five living in Lilongwe, Malawi^13^, and more broadly across Africa^14^, however these relationships have not been established among Malawian adults. In addition, malaria vectors have varying levels of resistance to different types of insecticide used on ITNs, such as alpha-cypermethrin, permethrin, and deltamethrin^15^, however, the effect of ITN insecticide type on malaria prevalence has not been assessed at the population level.

The objective of the current analysis is to characterize and identify changes in the prevalence of asymptomatic *P. falciparum*, due to demographic, environmental, and spatial risk factors in Malawian adolescents and adults ages 15–54 years. Using dried blood spots collected from the 2015–2016 Malawi Demographic and Health Survey (2015–2016 MDHS), we conducted quantitative polymerase chain reaction (PCR) to detect parasitemia. We then identified risk factors for infection using data taken from the 2015–2016 MDHS and other spatial and environmental information sources^16–18^. A particular focus was placed on ownership and self-reported use of bed nets. Understanding the prevalence and spatial distribution of underlying infection is essential for implementation and evaluation of future interventions. Molecular surveillance of *P. falciparum* in adults can be an important tool for Ministries of Health to supplement ongoing collection of clinical data through national health management information systems and periodic national household surveys of malaria prevalence among children.

## Methods

### Study design and population

The 2015–2016 MDHS was a cross-sectional, nationally-representative survey enrolling 26,361 households, 24,562 individual female participants, and 7478 individual male participants, from a total of 850 clusters between October 2015 and February 2016, during the annual transition between dry and rainy seasons^19^. In addition to household and individual interviews, women ages 15–49 and men ages 15–54 were also asked to contribute dried blood spots (DBS), collected on filter paper, to measure HIV prevalence. Of the 15,125 eligible individuals who contributed DBS for HIV testing, 7393 (48.9%) unique DBS cards were subsequently located and found to have enough sample remaining for use in the current study. Informed consent was obtained from all individuals and/or their parent or legal guardian for participation in the 2015–2016 MDHS, collection and storage of DBS, and additional testing of their samples. Ethical approval for our analysis was obtained from Institutional Review Boards through the National Health Sciences Research Committee at the Malawi Ministry of Health (#19/08/2381) and the University of North Carolina at Chapel Hill (#19–2882), and all research was performed in accordance with relevant guidelines and regulations.

### DNA amplification and genotyping

DBS were punched into 96 well plates at UNC Project-Malawi and shipped to the University of North Carolina at Chapel Hill for testing. DNA was extracted from filter paper using Chelex and stored at − 80 °C. Further details on the DNA extraction protocol and PCR assay validation are included in the Technical Methods found in the Supplemental Material. We tested each individual sample with a real time PCR assay targeting the *P.f. lactate de-hydrogenase* gene (*pfldh*) to identify individuals with *P. falciparum* malaria infection, our primary outcome^20^. Samples amplified with a PCR cycle threshold (C_T_) value above 39 were considered negative. Sensitivity analyses considered a range of C_T_ value cut-offs to determine the impact of altering the sensitivity of the assay (Supplementary Fig. 1).

### Spatial and ecological variables

De-identified 2015–2016 MDHS survey and geospatial data were linked to each sample’s PCR results through random sample barcode. As part of the DHS methodology, GPS coordinates are collected in the field, marking the center of each cluster of households. The DHS program maintains participant confidentiality by displacing the GPS coordinates for all survey clusters: urban clusters are displaced up to a maximum of 2 km and rural clusters up to 5 km, with an additional 1% subset of rural clusters displaced up to 10 km^21^. PCR *P. falciparum* prevalence was mapped onto a smoothed surface using a constructed semivariogram and simple kriging to predict regional variation in malaria prevalence. Simple kriging assumes that observed malaria prevalence is spatially autocorrelated and that there is a known mean trend which is stationary across our study area.

Individual and cluster level risk factors were selected based on directed acyclic graphs and known associations from relevant literature^7–9,22,23^. Individual level factors included sex, age group, wealth quintile, education level, owning livestock, source of drinking water, living in a household with a bed net, sleeping under a long-lasting insecticide-treated net (LLIN), LLIN insecticide type, living in a household with at least 1 net per 1.8 household members, and anemia (women only). Cluster level covariates included region, urban/rural place of residence, elevation, month of data collection, landcover, the proportion of a cluster with bed nets, and the proportion of a cluster that slept under an LLIN. As transmission intensity is seasonal in Malawi, with peak transmission between January and May due to greater rainfall^11,24^, we also examined environmental variables at the cluster level including current month’s average daily maximum temperature, and the prior month’s precipitation. Modeled monthly precipitation and monthly average daily maximum temperature raster files, created from both in-situ weather station data and satellite imagery, were acquired from the Climate Hazards Center at the University of California, Santa Barbara^16,17^. Clusters were assigned precipitation and temperature values by averaging raster cells which fell within the 2 km and 10 km buffers surrounding urban and rural clusters, respectively, similar to DHS methodology for ecological variables^25^. Land cover estimates were obtained from satellite imagery classified by the Regional Center for Mapping of Resources for Development and SERVIR-Eastern and Southern Africa into settlement areas, forest, grassland, cropland, wetland, and other land cover types^18^. Clusters were assigned the majority land cover value within the 2 km and 10 km buffers surrounding urban and rural clusters.

### Statistical analysis

We estimated prevalence differences to quantify the associations between demographic and environmental risk factors and the probability of being infected with *P. falciparum*. To power findings within subgroups of interest, the DHS two-stage sampling design selects population clusters with unequal probability, followed by households within clusters, violating the independence of observations assumption in standard regression. Weighting individual observations is required to appropriately specify individual and group-level variation. We accounted for the 2015–2016 MDHS complex sample survey design when estimating bivariate and multivariate associations using 2015–2016 MDHS HIV sample weights incorporated into linear risk regression models fit using generalized estimating equations. As the subset of DBS used in this analysis was not a simple random sample (Supplementary Table 4), we also incorporated standardized inverse probability of selection weights^26^ into regression models by calculating stabilized propensity scores for the probability of each individual being selected into the analysis based on their respective covariate pattern. Bivariate models were run between all risk factors and the malaria prevalence outcome, to determine high-risk populations and areas for targeted interventions. Multivariate adjusted analyses were performed to assess the association between bed net use, insecticide type, and malaria prevalence, after controlling for confounding variables, as bed nets have the potential for use in future interventions. Multivariate models were run among the total study population and in a sub-analysis of pregnant women. All weighted tabulations, models, and maps were run using R 3.6.2 (R Foundation for Statistical Computing, Vienna, Austria), using the *survey* (*v3.35–1*; Lumley, 2019) and *sf* (*v0.9–2*; Pebesma, 2020) packages.

## Results

**Figure 1:**
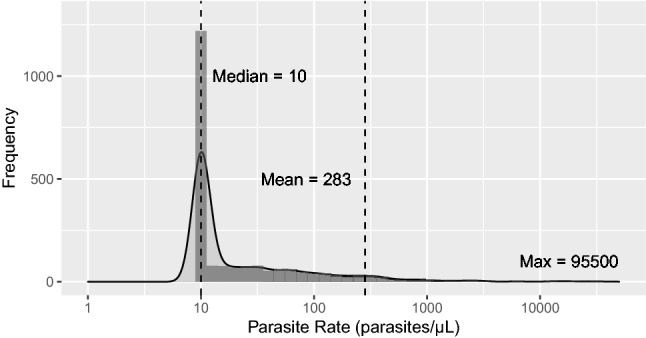
Distribution of PCR *P. falciparum* positive parasitemia values (n = 2215). Values < 10 parasites/µL are rounded up to 10 parasites/µL. The density plot’s solid line represents a normal distribution using the observed counts.

Of all 7393 samples analyzed, 2,125 were PCR-positive for *P. falciparum*, and 5268 were negative. After incorporating survey weights, the weighted *P. falciparum* prevalence was 31.1% (standard error = 1.1) among adolescents and adults ages 15–54 (Table 1). Samples represented 497 out of 850 (58.5%) clusters. The median number of individuals per cluster was 16 (IQR 12 to 30; range 1 to 34). Over half of positive samples (55.6%) had parasitemias ≤ 10 parasites/µL (Fig. 1), and parasitemia was not correlated with age. The intra-class coefficient at the cluster level was 22.3%, indicating that individual risk is associated with cluster-level risk.

**Table 1:** Characteristics of the study population, stratified by *P. falciparum* PCR status, 2015–2016 Malawi Demographic and Health Survey.

	Variable	PCR-negative		PCR-positive		Total	
		n	%	n	%	n	%
Unweighted total number*		5268		2125			
Weighted count proportion**		4799	68.9	2170	31.1	6969	
Individual level covariates**							
Sex	Male	2191	45.7	1124	51.8	3315	47.6
	Female	2608	54.3	1045	48.2	3654	52.4
Age group (years)	15–24	1936	40.3	1096	50.5	3031	43.5
	25–34	1421	29.6	583	26.9	2004	28.8
	35–44	1004	20.9	349	16.1	1353	19.4
	45–54	439	9.1	142	6.5	581	8.3
Wealth quintiles	Poorest	712	14.8	530	24.4	1242	17.8
	Poorer	849	17.7	524	24.1	1372	19.7
	Middle	943	19.6	490	22.6	1433	20.6
	Richer	1004	20.9	366	16.9	1370	19.7
	Richest	1291	26.9	260	12.0	1551	22.3
Education	None/preschool	373	7.8	186	8.6	559	8.0
	Primary	2816	58.7	1525	70.3	4341	62.3
	Secondary	1381	28.8	442	20.4	1823	26.2
	Higher	209	4.4	12	0.5	221	3.2
Owns livestock, herds, or farm animals	No	2207	46.0	946	43.6	3153	45.2
	Yes	2593	54.0	1224	56.4	3816	54.8
Source of drinking water	Piped	1222	25.5	250	11.5	1472	21.1
	Unpiped	3577	74.5	1920	88.5	5497	78.9
Household has a bed net	No	1606	33.5	749	34.5	2355	33.8
	Yes	3193	66.5	1421	65.5	4614	66.2
Slept under an LLIN last night	No	3014	62.8	1408	64.9	4422	63.4
	Yes	1786	37.2	762	35.1	2547	36.6
Insecticide of LLIN individual slept under (out of individuals sleeping under nets)	Permethrin	1141	63.9	507	66.6	1649	64.7
	Non-permethrin	642	36.0	254	33.4	897	35.2
At least 1 net per 1.8 household members	No	731	15.2	243	11.2	975	14.0
	Yes	2462	51.3	1178	54.3	3640	52.2
Anemia (women only)	Not anemic	1821	69.8	628	60.1	2449	67.0
	Mild	601	23.1	327	31.3	929	25.4
	Moderate	173	6.6	81	7.8	254	7.0
	Severe	10	0.4	9	0.9	19	0.5
Cluster level covariates**							
Region	Northern	822	17.1	254	11.7	1076	15.4
	Central	1653	34.4	914	42.1	2567	36.8
	Southern	2325	48.4	1001	46.1	3326	47.7
Place of residence	Urban	941	19.6	152	7.0	1093	15.7
	Rural	3858	80.4	2018	93.0	5876	84.3
Elevation (m)	< 500	719	15.0	287	13.2	1005	14.4
	≥ 500 & < 1000	1673	34.9	1006	46.4	2678	38.4
	≥ 1000 & < 1500	2217	46.2	849	39.2	3067	44.0
	≥ 1500	191	4.0	28	1.3	219	3.1
Month of data collection	October '15	867	18.1	635	29.3	1503	21.6
	November '15	1580	32.9	639	29.5	2219	31.8
	December '15	730	15.2	234	10.8	963	13.8
	January '16	1462	30.5	568	26.2	2029	29.1
	February '16	161	3.4	94	4.3	255	3.7
Landcover	Settlement	819	17.1	116	5.4	935	13.4
	Forest	605	12.6	313	14.4	917	13.2
	Grassland	333	6.9	185	8.5	518	7.4
	Cropland	2743	57.1	1410	65.0	4153	59.6
	Wetland	220	4.6	131	6.0	350	5.0
	Other	80	1.7	15	0.7	96	1.4

		Mean	SE	Mean	SE	Mean	SE
Proportion of cluster with bed nets		64.4	0.9	63.6	1.1	64.2	0.8
Proportion of cluster that slept under an LLIN last night		31.5	0.7	30.6	0.8	31.2	0.6
Current month's average daily maximum temperature (°C)		31.1	0.1	31.5	0.1	31.2	0.1
Prior month's precipitation (mm)		67.3	4.0	62.3	4.7	65.7	3.9

*Counts do not incorporate sample weights, and are not representative of the weighted populations used in the table.

**Sampling weights applied.

*LLIN* long-lasting insecticide-treated net.

### Demographics

After incorporating weights to account for survey design and selection bias, 52.4% of participants were female, 43.5% were between the ages of 15 to 24, and primary school was the highest level of education attended by 62.3% (Table 1). Most participants came from the Central (36.8%) and Southern regions (47.7%), and 84.3% were from rural areas. Most individuals (59.6%) were from clusters located on cropland. Data collection was spread unevenly across months, ranging from 3.7% of samples collected in February 2016 to 31.8% in November 2015. Two-thirds (66.2%) of participants lived in a household with a bed net, but only 36.6% reported sleeping under a long-lasting insecticide-treated bed net (LLIN) the night prior; 64.7% under permethrin treated LLINs and 35.2% under non-permethrin (alpha-cypermethrin or deltamethrin) treated LLINs. Over half (52.2%) of individuals lived in households meeting the World Health Organization’s universal coverage criteria of a minimum of 1 net per 1.8 people.

**Figure 2:**
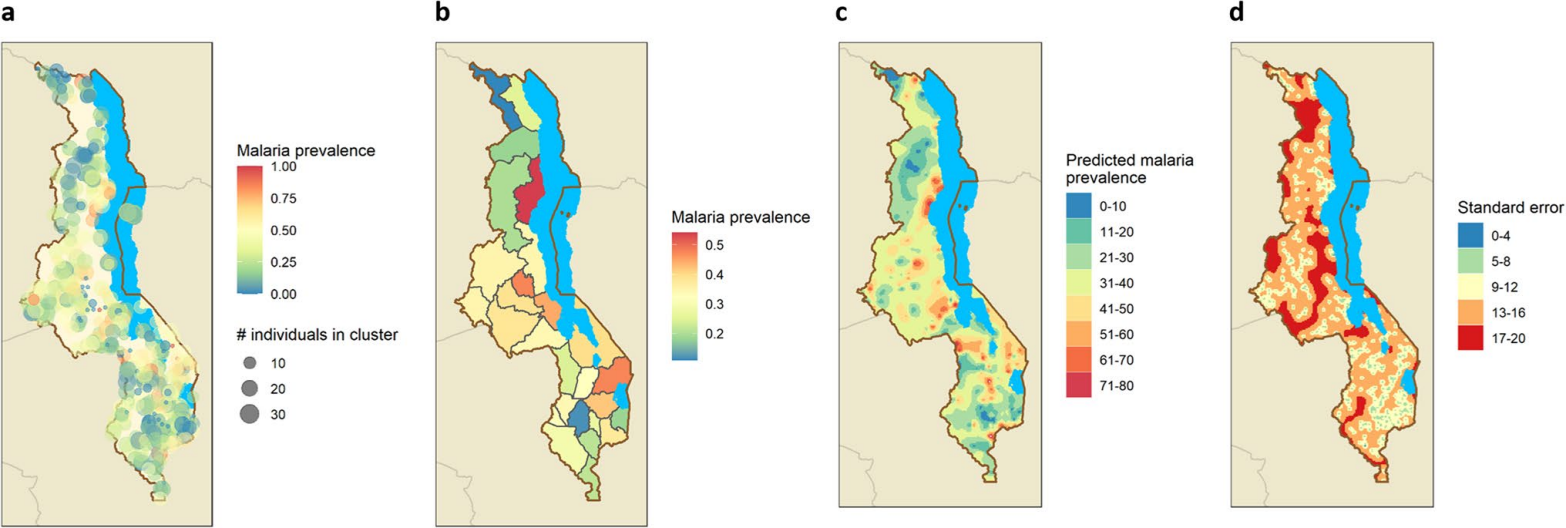
Spatial distribution of 2015–2016 MDHS clusters. (**a**) *P. falciparum* prevalence by cluster and cluster size, (**b**) weighted *P. falciparum* prevalence by district, (**c**) smoothed PCR *P. falciparum* prevalence estimates using simple kriging, (**d**) smoothed *P. falciparum* standard error estimates using simple kriging. Clusters with fewer than five observations were removed prior to kriging to reduce the influence of extreme values due to small sample sizes. Smoothed surfaces are meant to demonstrate regional differences and should not be used for interpretation into areas where data do not exist. All maps were run using R 3.5.1 (R Foundation for Statistical Computing, Vienna, Austria) and the *sf* (*v0.9–2*; Pebesma, 2020) package.

Simple kriging *P. falciparum* prevalence estimates ranged from 2.5 to 83.5% across Malawi, demonstrating high spatial heterogeneity (Fig. 2). *P. falciparum* prevalence was higher in the Northeastern part of the country along the lakeshore, and in the central southern region near Ntcheu. Prevalence was lowest in the southern tip of Malawi and in the Northwest highland areas. Estimates should be interpreted regionally, as standard errors are high in areas where data do not exist.

### Risk factor analysis

In weighted bivariate analysis, female sex was associated with a lower prevalence of *P. falciparum* infection (prevalence difference − 0.05, 95% confidence intervals − 0.08 to − 0.03) (Table 2). Prevalence decreased as age increased and was lowest among those 45–54 years as compared to those 15–25 years (PD − 0.12, 95% CI − 0.17 to − 0.06). Parasite prevalence was lower in urban versus rural areas (PD − 0.20, 95% CI − 0.24 to − 0.17) and among populations with piped drinking water versus unpiped (PD − 0.18, 95% CI − 0.22 to − 0.14). Other protective factors included greater wealth (PD − 0.26, 95% CI − 0.31 to − 0.21, richest vs. poorest), higher education (PD − 0.28, 95% CI − 0.34 to − 0.22, higher vs. none). Living in wetlands (PD 0.25, 95% CI 0.15 to 0.34) and grasslands (PD 0.23, 95% CI 0.13 to 0.33) were associated with the highest prevalence of infection, as compared to settlement areas. Increased geographic average daily maximum temperature was associated with greater malaria prevalence (PD 0.02, 95% CI 0.01 to 0.03 per 1 °C increase). Individuals living at elevations between 500 and 1000 m above sea level, along the lake shore, had the highest prevalence of infection (37.6%), with lowest prevalence among those living at or above elevations of 1500 m above sea level (12.7%), although other geographic factors likely contributed to variations in estimates.

**Table 2:** Bivariate associations between demographic and environmental risk factors and *P. falciparum* prevalence using weighted survey data.

Covariates	Variable	*P. falciparum* prevalence	Prevalence difference	95% confidence interval		p-value
Sex	Male	0.339	–	–	–	–
	Female	0.286	− 0.05	− 0.08	− 0.03	< 0.001
Age group (years)	15–24	0.361	–	–	–	–
	25–34	0.291	− 0.07	− 0.11	− 0.04	< 0.001
	35–44	0.258	− 0.10	− 0.15	− 0.06	< 0.001
	45–54	0.245	− 0.12	− 0.17	− 0.06	< 0.001
Wealth quintiles	Poorest	0.427	–	–	–	–
	Poorer	0.382	− 0.05	− 0.09	0.00	0.05
	Middle	0.342	− 0.08	− 0.14	− 0.03	0.001
	Richer	0.267	− 0.16	− 0.20	− 0.11	< 0.001
	Richest	0.167	− 0.26	− 0.31	− 0.21	< 0.001
Education	None	0.332	–	–	–	–
	Primary	0.351	0.02	− 0.03	0.07	0.4
	Secondary	0.243	− 0.09	− 0.14	− 0.04	0.001
	Higher	0.053	− 0.28	− 0.34	− 0.22	< 0.001
Owns livestock, herds or farm animals	No	0.300	–	–	–	–
	Yes	0.321	0.02	− 0.01	0.05	0.2
Source of drinking water	Piped	0.170	–	–	–	–
	Unpiped	0.349	0.18	0.14	0.22	< 0.001
Household has a bed net	No	0.318	–	–	–	–
	Yes	0.308	− 0.01	− 0.04	0.02	0.6
Slept under an LLIN last night	No	0.318	–	–	–	–
	Yes	0.299	− 0.02	− 0.05	0.01	0.2
Insecticide of LLIN individual slept under (out of individuals sleeping under nets)	Permethrin	0.308	–	–	–	–
	Non− permethrin	0.284	− 0.02	− 0.08	0.03	0.4
At least 1 net per 1.8 household members	No	0.249	–	–	–	–
	Yes	0.324	0.07	0.03	0.12	0.001
Number of months ago that sleeping net was obtained (treated or untreated)	< 12	0.302	–	–	–	–
	12–23	0.294	− 0.01	− 0.07	0.05	0.8
	24–35	0.281	− 0.02	− 0.10	0.06	0.6
	≥ 36	0.265	− 0.04	− 0.09	0.02	0.2
Anemia (women only)	Not anemic	0.256	–	–	–	–
	Mild	0.352	0.10	0.05	0.14	< 0.001
	Moderate	0.319	0.06	− 0.01	0.14	0.1
	Severe	0.478	0.22	− 0.05	0.49	0.1
Region	Northern	0.236	–	–	–	–
	Central	0.356	0.12	0.07	0.17	< 0.001
	Southern	0.301	0.06	0.01	0.12	0.02
Place of residence	Urban	0.139	–	–	–	–
	Rural	0.343	0.20	0.17	0.24	< 0.001
Elevation (m)	< 500	0.285	–	–	–	–
	≥ 500 & < 1000	0.376	0.09	0.03	0.16	0.007
	≥ 1000 & < 1500	0.277	− 0.01	− 0.07	0.05	0.8
	≥ 1500	0.127	− 0.16	− 0.23	− 0.09	0.000
Month of data collection	October '15	0.423	–	–	–	–
	November '15	0.288	− 0.13	− 0.21	− 0.06	< 0.001
	December '15	0.243	− 0.18	− 0.26	− 0.10	< 0.001
	January '16	0.280	− 0.14	− 0.21	− 0.08	< 0.001
	February '16	0.369	− 0.05	− 0.16	0.06	0.3
Landcover	Settlement	0.124	–	–	–	–
	Forest	0.341	0.22	0.14	0.29	< 0.001
	Grassland	0.356	0.23	0.13	0.33	< 0.001
	Cropland	0.340	0.22	0.18	0.25	< 0.001
	Wetland	0.373	0.25	0.15	0.34	< 0.001
	Other	0.160	0.04	0.00	0.07	0.03
Proportion of cluster with bed nets (scaled)	Mean	0.311	–	–	–	–
	10% increase	–	− 0.01	− 0.02	0.01	0.4
Proportion of cluster that slept under an LLIN last night (scaled)	Mean	0.311	–	–	–	–
	10% increase	–	− 0.01	− 0.03	0.00	0.2
Current month's average daily maximum temperature (°C) (scaled)	Mean	0.311	–	–	–	–
	1 °C increase	–	0.02	0.01	0.03	< 0.001
Prior month's precipitation (mm) (scaled)	Mean	0.311	–	–	–	–
	100 mm increase	–	− 0.02	− 0.05	0.01	0.2

*LLIN* long-lasting insecticide treated net.

After adjusting for age, sex, wealth, and household size, there was no difference in the prevalence of infection between households with and without bed nets (PD 0.02, 95% CI − 0.02 to 0.05) or between individuals who reported sleeping under an LLIN the previous night and those who did not (PD 0.01, 95% CI − 0.02 to 0.04) (Table 3). After adjusting for age, sex, and wealth, sleeping under a non-permethrin versus permethrin treated LLIN (PD − 0.02, 95% CI − 0.07 to 0.02) was not protective. However, a sub-analysis among the study population’s 319 pregnant women showed a protective prevalence difference between those who slept under non-permethrin treated LLINs, including alpha-cypermethrin and deltamethrin, (PD − 0.16, 95% CI − 0.34 to 0.02) as compared to permethrin treated LLINs (Table 4), although this did not reach statistical significance. Meeting the WHO universal coverage criteria of 1 net per 1.8 household members was not protective in either population. Community-level household bed net coverage and the community-level proportion who slept under an LLIN were not protective against *P. falciparum* infection in the general population.

**Table 3:** Multivariate associations between bed net associated risk factors and *P. falciparum* prevalence using weighted survey data.

Exposure	Model	Prevalence difference	95% confidence interval		p-value
Household has a bed net	Unadjusted	− 0.01	− 0.04	0.02	0.6
	Adjusted*	0.02	− 0.02	0.05	0.3
Slept under an LLIN last night	Unadjusted	− 0.02	− 0.05	0.01	0.2
	Adjusted*	0.01	− 0.02	0.04	0.6
Individual slept under LLIN treated with non-permethrin (vs. permethrin)	Unadjusted	− 0.02	− 0.08	0.03	0.4
	Adjusted^†^	− 0.02	− 0.07	0.02	0.4
At least 1 net per 1.8 household members	Unadjusted	0.07	0.03	0.12	< 0.001
	Adjusted^§^	0.02	− 0.01	0.06	0.2

*LLIN* long-lasting insecticide treated net.

*Models adjusted for age, sex, wealth, and household size.

^†^Model adjusted for age, sex, and wealth.

^§^Model adjusted for wealth and district.

**Table 4:** Multivariate associations between bed net associated risk factors and *P. falciparum* prevalence using weighted survey data among pregnant women in the 2015–2016 MDHS who contributed samples to the analysis (n = 319).

Exposure	Model	Prevalence difference	95% confidence interval		p-value
Household has a bed net	Unadjusted	− 0.09	− 0.21	0.03	0.2
	Adjusted*	− 0.09	− 0.21	0.03	0.1
Slept under an LLIN last night	Unadjusted	− 0.06	− 0.20	0.07	0.4
	Adjusted*	− 0.06	− 0.19	0.08	0.4
Individual slept under LLIN treated with non-permethrin (vs. permethrin)	Unadjusted	− 0.19	− 0.37	− 0.02	0.04
	Adjusted^†^	− 0.16	− 0.34	0.02	0.08
At least 1 net per 1.8 household members	Unadjusted	0.02	− 0.15	0.18	0.8
	Adjusted^§^	0.03	− 0.09	0.15	0.6

*LLIN* long-lasting insecticide treated net.

*Models adjusted for age, wealth, and household size.

^†^Model adjusted for age and wealth.

^§^Model adjusted for wealth and region.

Sensitivity analyses defining malaria positivity as PCR amplification which crossed the threshold line below C_T_ values of 37 and 38 found similar relationships between covariates and *P. falciparum* prevalence in bivariate and multivariate analyses (Supplementary Tables 5–8).

## Discussion

This study represents the first national survey in Malawi to determine *P. falciparum* infection in adolescents and adults. Nearly a third of individuals ages 15–54 are infected with *P. falciparum*, primarily with low-density infections undetectable through microscopy or RDT. Protective factors against asymptomatic *P. falciparum* infection include older age, urban residence, greater wealth, higher education, and lower geographic average daily maximum temperature. Living in a household which owned a bed net and reporting sleeping under an LLIN were not protective against infection among our study population, even after stratifying by insecticide type. However, among pregnant women, sleeping under alpha-cypermethrin or deltamethrin-treated nets appeared protective, as compared to permethrin treated nets. Underappreciating the significance of the extensive reservoir of asymptomatic infection among adolescents and adults neglects an important source of sustained *P. falciparum* transmission in Malawi and hinders understanding of key target groups for intervention.

Demographic and environmental risk factors associated with *P. falciparum* infection among adolescent and adults in our study resemble those previously found among asymptomatic children and individuals of all ages presenting with clinical symptoms; these risk factors in Malawi include low elevation, higher temperatures, younger age, rurality, and region^7–9^. Similar to the cross-sectional nationally representative Malawi Malaria Indicator Surveys (MIS) among children in 2014 and 2017^27,28^, the highest malaria prevalence was found in the Central region, followed by the Southern and Northern regions, although results are likely influenced by regional data collection during different months of the year (Supplementary Table 9). The national prevalence estimate of 31% in our study is comparable to results using similar methodology among adults from the Democratic Republic of the Congo in 2007 and 2014, which also found that younger age, male sex, and lower wealth indicators were risk factors for increased infection^29,30^. Our prevalence estimate is much higher than modeling predictions of *P. falciparum* annual parasite rates of 18–19% in children 2–10 years in 2015–2016, however these predictions were generated from multiple community-based survey measurements in the literature and from other unpublished sources which used RDT and microscopy as opposed to PCR^6^. While prevalence estimates were high, parasitemia values were low among our study population; however this finding is consistent with research showing that malaria infection is more likely to be submicroscopic among older children and adults versus younger children, hypothesized to stem from acquired immunity^31^. Risk factor analysis is important for identifying key populations to target for malaria prevention and control, and our results suggest that malaria prevention measures might be best focused towards men, younger age groups, poor communities, and rural areas.

Surprisingly, household ownership of bed nets, individual use of LLINs for sleeping, owning 1 net per 1.8 household members, and community bed net coverage were not associated with asymptomatic *P. falciparum* prevalence. While two-thirds of individuals resided in households which owned bed nets, only 36.6% reported sleeping under an LLIN the previous night, indicating low LLIN usage. Prevalence was higher among men as compared to women; females have been shown to clear asymptomatic *P. falciparum* infections faster than males, leading to the appearance of lower infectivity, and highlighting the importance of biological differences^32^. Other reasons for prevalence differences between males and females could relate to gender norms and behavioral factors around sleeping arrangements, gender-differentiated access to education about malaria or treatment and screening services, and gendered division of outdoor labor^33^.

Bed net ownership and use indicators were not associated with *P. falciparum* prevalence among our overall study population but insecticide type was found to have a near protective association among pregnant women. Pregnant women had similar patterns of sleeping under an LLIN the previous night as compared to the overall study population (38.7% vs. 36.6% respectively), however of those who slept under LLINs, 43.6% of pregnant women used nets treated with alpha-cypermethrin or deltamethrin, compared with 35.2% in the general population. Additionally, 49.0% of the nets used by pregnant women for sleeping were less than one year old, compared with 37.2% in the general population. While the general population receives bed nets every three years as part of regular mass distribution campaigns, Malawi’s National Malaria Control Programme has given out free LLINs to pregnant women through antenatal care clinics since 2006^11^ with 79–87% of pregnant women who attended antenatal clinics receiving LLINs in 2015–16^11^. Although WHO recommendations allow for up to three years between mass distribution campaigns^34^, field research in Benin, Malawi, Rwanda, Senegal, and Tanzania suggests that ITNs have a limited lifespan of two years before protection is compromised by holes, insecticide-resistance, and reduced concentrations of insecticide^35–39^. In Burkina Faso, a third of people had stopped using LLINs within a year of distribution^40^. Pregnant women in our study appear to have been the recipients of relatively newer nets which might have greater effectiveness, while households without pregnant women would have primarily received nets through Malawi’s first national mass distribution campaign in 2012, or through limited follow-up mop-up campaigns in six districts in 2014^41^. In areas where nets are used at night, there is also evidence to suggest that long term use of insecticides inside the home can shift *Anopheles spp.* mosquitos from indoors to outdoors host seeking behavior, increasing the likelihood of an individual becoming infected, and perhaps contributing to reduced effectiveness of LLINs against malaria^42,43^. Bed net durability and mosquito biting behavior were not measured as part of the 2015–2016 MDHS, but could have contributed to the lack of association found between net ownership and use, and malaria prevalence among our study population.

The major strength of this study is the efficient use of a large number of nationally collected samples from adolescents and adults, a population which is understudied in malaria transmission research. Using molecular and epidemiologic methods, we better characterized the reservoir of asymptomatic *P. falciparum*, a pool of infection which is likely contributing to sustained transmission in Malawi. The results presented can serve as baseline assessment; repeated use of DHS samples over time can create a picture of shifting trends across the entire country while using resources cost-effectively to supplement intermittent MIS iterations estimating malaria prevalence among children. Characterizing the prevalence of *P. falciparum* among adults and identifying key target groups will be informative as the Malawi Ministry of Health designs future mass distribution campaigns and other interventions against malaria.

The primary limitation of this analysis is the cross-sectional nature of available samples. The 2015–2016 MDHS captured DBS from participants at a single time point, allowing for estimation of marginal associations between risk factors and infection to identify groups at high risk, but limiting evaluation of causal relationships. Additionally, the samples used in the study were collected during the 2015–2016 MDHS survey period from October to February, limiting inference to the remainder of the year and hindering comparison with published prevalence estimates among children from different time periods; however, as the study period occurred during the transition from dry to rainy season, we anticipate that results are somewhat representative of a yearly average. We used inverse probability of selection weights to make our results generalizable to the broader DHS cohort, but additional bias could still result due to unmeasured confounding. Our analysis was also constrained by the aggregated geographical classification of individuals. As part of DHS methodology, individuals are geolocated at the center of their study cluster, which is then displaced up to 5 km for 99% of rural clusters and 2 km for urban clusters. There is an inherent lack of precision in land cover, temperature, and precipitation data, nonetheless, our methods attempt to account for geographic displacement by using average values falling within a cluster’s potential buffer area. Although our study measures presence of *P. falciparum*, we were not able to ascertain the presence of gametocytes within each infection, limiting the extent to which we can predict how these infections continue to sustain transmission. Results from elsewhere in Africa show that infection with *P. falciparum* gametocytes is associated with low asexual parasite densities and asymptomatic disease^3^. Comparisons between microscopy and PCR have found that microscopy can miss over 90% of gametocyte carriers due to limited sensitivity for low-density infections^44^, further highlighting the importance of molecular surveillance tools in understanding infection transmission dynamics in this population.

Despite existing limitations, this analysis provides valuable input into an understudied yet critical group to consider in efforts to interrupt ongoing malaria transmission. Malawi spent an estimated $82 million on malaria control in 2016^45^ and malaria accounts for 30% of all outpatient visits and 34% of inpatient hospital admissions^11^. Households often amass high direct and indirect costs due to clinical malarial disease, despite free diagnosis and treatment^46^. One of the primary goals of the Malawi Malaria Strategic Plan 2017–2022 is to achieve universal LLIN coverage for all households^11^. Research from Madagascar shows that while LLIN mass distribution campaigns may only provide community protection for one year, protection can be sustained when campaigns are followed by continuous LLIN distribution to eligible households, including recently married couples, immigrants, children of vaccination age, and homes with uncovered sleeping areas^47^. Malawi could benefit from education on consistent and correct use of bed nets, and through expansion of continuous LLIN distribution services to additional populations beyond pregnant women, targeting younger individuals living in rural areas with high prevalence of infection for more frequent net replacement. Treating malaria at the population level through mass drug administration can clear parasite presence and prevent transmission of gametocytes from asymptomatic infections. However, mass drug administration is only recommended in settings considering malaria elimination, and requires low malaria prevalence, effective vector control, access to treatment, and extensive community participation as prerequisites for implementation^48,49^.

This study presents unique insight into the national prevalence of asymptomatic *P. falciparum* infection among adolescent and adults in Malawi. Use of molecular and epidemiological surveillance methods in tandem demonstrates that demographic and environmental risk factors for infection parallel those found in children and among individuals with symptomatic disease. Within the current framework of mass distribution frequency and community education, presence of bed nets in the household and use of LLINs by the individual did not appear to provide protective benefits, regardless of insecticide type, most likely due to bed net age and low frequency of use. Results from this study provide valuable guidance to decision makers in Malawi as the National Malaria Control Programme designs bed net distribution programs following mid-term review of the 2017–2022 National Malaria Control Strategy. Future work to replicate this analysis following the 2021–2022 MDHS will enable assessment of changes in asymptomatic *P. falciparum* prevalence and other genetic markers in adolescents and adults across time.

## Supplementary information

Supplementary Information 1.

Supplementary Information 2.

## Data Availability

All relevant data are within the manuscript and supplement. Data that support study findings are available for download from the DHS MEASURE website, conditional on approval from DHS. Laboratory testing data are available from the authors upon reasonable request.
