# Supplementary material for: Asymptomatic Plasmodium falciparum malaria prevalence among adolescents and adults in Malawi, 2015–2016
Source: Sci Rep. 2020 Oct 30;10:18740. doi: 10.1038/s41598-020-75261-9 (PMC7603306; doi:10.1038/s41598-020-75261-9)
Supplement: Supplementary file 2 — Supplementary Information 2. [file 41598_2020_75261_MOESM2_ESM.docx]

**SUPPLEMENTAL MATERIAL**

**Hidden Reservoir: Asymptomatic *Plasmodium falciparum* Malaria Prevalence among Adolescents and Adults in Malawi, 2015-2016**

**Authors**: Hillary M. Topazian, Austin Gumbo, Sydney Puerto-Meredith, Ruth Njiko, Alexis Mwanza, Michael Kayange, David Mwalilino, Bernard Mvula, Gerald Tegha, Tisungane Mvalo, Jessie K. Edwards, Michael Emch, Audrey Pettifor, Jennifer S. Smith, Irving Hoffman, Steven R. Meshnick, Jonathan J. Juliano

**TECHNICAL METHODS**

**DNA amplification and genotyping**

DBS were punched in triplicate into 96 well plates using a 6 mm hole-punch at UNC Project-Malawi and shipped to the University of North Carolina at Chapel Hill for testing. DBS samples were stored at -20°C prior to parasite DNA extraction using a Tween-Chelex protocol modified from Teyssier et al. 2019. One milliliter of 0.5% Tween 20 in 1X PBS was added to each well and plates were incubated at room temperature overnight on shakers. After centrifuging, Tween-PBS was aspirated, and samples were washed with 1 mL of 1X PBS and underwent a 15-30-minute incubation at 4°C. After centrifuging again, PBS was aspirated and 150 µL of a 1:2 solution of 20% Chelex 100 resin in water was added to each well and plates were incubated for 10 minutes at 95°C, occasionally shaking. Plates were centrifuged at 1,500 rmp for 10 minutes and supernatant was transferred to non-skirted 96 well plates and centrifuged again at 1,500 rpm for 5 minutes, removing the supernatant to an Eppendorf LoBind DNA 96-well plate for storage. DNA was extracted and stored at -80°C until qPCR.

Samples were run in singular in 384 well plates alongside 2 negative controls and 2 of each concentration of positive controls across a wide range of parasitemias: 100,000; 1,000; 100; 10; and 1 parasite/µL. PCR primers and probe are noted in Supplementary Table 1. Real time PCR was conducted in 12 µL volumes, using Roche Universal Probe Master Mix and 2 µL of input DNA. The PCR cycles were 50C for 2 minutes and 95C for 10 minutes, followed by 40 cycles of 95C denaturation for 15 seconds and 60C annealing/extension for 1 minute. Parasitemias were calculated using a trendline based on the Ct values of the positive control standards within each plate.

**PCR assay validation**

In total we conducted 908 control reactions, including 480 positive controls and 428 negative controls (Supplementary Table 2). Negative controls were either water (n=48) or human DNA (0.1ng/µL) from a non-exposed population (n=380). Positive controls were generated across a range of parasitemias, from 10,000 parasites/µL to 1 parasite/µL. Cultured parasites (strain 3D7, MRA-102, BEI Resources, Manassas VA) were quantified using repeated counts on a hemocytometer and mixed with human whole blood in order to mock desired parasitemias. The spiked blood was dried onto filter paper at 70 µL per spot, and DNA was extracted from these filter papers using the same methodology as the clinical samples. Extracted spiked blood DNA was used for all control reactions and would approximate any losses of DNA through the extraction process. We conducted a higher number of replicates at the lowest parasitemia value (n=194) to mimic the expected distribution of parasitemias in the clinical samples. All assay results and amplification curves were manually evaluated on the machine prior to exportation to the database. Based on these data, we estimated the sensitivity, specificity, negative predictive value (NPV) and positive predictive values (PPV) across a range of prevalence estimates (Supplementary Table 3). Samples amplified with a PCR cycle threshold (CT) value above 39 were considered negative in the final analysis to remain conservative in our classification of *P. falciparum* infected individuals. A 39-cycle cutoff is approximately one standard deviation above the mean Ct value for our 194 replicates of 1 parasite/µl, our lowest positive control standard. The resultant data suggests that the assay has an extremely low false positivity rate.

Reference:

Teyssier, N.B. *et al.* Optimization of whole-genome sequencing of *Plasmodium falciparum* from low-density dried blood spot samples. *bioRxiv*. (2019)

**SUPPLEMENTARY TABLES**

**Supplementary Table 1**. Primers and reaction conditions; each reaction consisted of 12 µL.

| factor | Final Concentration | sequence |
| --- | --- | --- |
| Forward primer | 300 nM | ACGATTTGGCTGGAGCAGAT |
| Reverse primer | 300 nM | TCTCTATTCCATTCTTTGTCACTCTTTC |
| Probe | 250 nM | FAM-AGTAATAGTAACAGCTGGATTTACCAAGGCCCCA-TAMRA |

**Supplementary Table 2**. Control reactions. Negative controls represent water (n=48) or human DNA (n=380).

| Parasites/µL | N | # positive at Ct 39 | % positive at Ct 39 | Mean Ct | Median Ct | Standard deviation Ct |
| --- | --- | --- | --- | --- | --- | --- |
| 0 | 428 | 0 | 0 | - | - | - |
| 1 | 194 | 148 | 76.3 | 37.5 | 37.1 | 1.61 |
| 5 | 48 | 48 | 100 | 34.3 | 34.3 | 0.34 |
| 10 | 84 | 83 | 98.8 | 33.7 | 33.4 | 1.12 |
| 100 | 50 | 50 | 100 | 30.8 | 30.6 | 1.25 |
| 1,000 | 52 | 52 | 100 | 26.5 | 26.3 | 0.89 |
| 10,000 | 52 | 52 | 100 | 22.6 | 22.6 | 0.63 |

**Supplementary Table 3**. Sensitivity, specificity, negative predictive value, and positive predictive values across a range of *P. falciparum* prevalence estimates.

| prevalence | Sensitivity | specificity | positive predictive value | negative predictive value |
| --- | --- | --- | --- | --- |
| - | 90.2 | 100 | - | - |
| 0.10 | - | - | 100 | 98.9 |
| 0.20 | - | - | 100 | 97.6 |
| 0.30 | - | - | 100 | 96.0 |
| 0.40 | - | - | 100 | 93.9 |

**Supplementary Table 4**. Characteristics of 2015-16 MDHS samples which were available for the current analysis and those which were unavailable.

| **Variable** | | **Unavailable samples n (%)** | **Available samples n (%)** | **p-value*** |
| --- | --- | --- | --- | --- |
| **Total** |  | 7732 | 7393 |  |
|  |  |  |  |  |
| **# clusters** |  | 633 (74.5) | 497 (58.5) |  |
|  |  |  |  |  |
| **Sex** | Male | 3569 (46.2) | 3472 (47.0) | 0.3 |
|  | Female | 4163 (53.8) | 3921 (53.0) |  |
|  |  |  |  |  |
| **Age group** | 15-24 | 3257 (42.1) | 3285 (44.4) | 0.03 |
|  | 25-34 | 2312 (29.9) | 2093 (28.3) |  |
|  | 35-44 | 1554 (20.1) | 1429 (19.3) |  |
|  | 45-54 | 609 (7.9) | 586 (7.9) |  |
|  |  |  |  |  |
| **Wealth quintiles** | Poorest | 1273 (16.5) | 1100 (14.9) | <0.001 |
|  | Poorer | 1454 (18.8) | 1338 (18.1) |  |
|  | Middle | 1536 (19.9) | 1330 (18.0) |  |
|  | Richer | 1639 (21.2) | 1471 (19.9) |  |
|  | Richest | 1830 (23.7) | 2154 (29.1) |  |
|  |  |  |  |  |
| **Education** | None | 610 (7.9) | 548 (7.4) | 0.001 |
|  | Primary | 4701 (60.8) | 4336 (58.7) |  |
|  | Secondary | 2129 (27.5) | 2244 (30.4) |  |
|  | Higher education | 277 (3.6) | 238 (3.2) |  |
|  | Missing | 15 (0.2) | 27 (0.4) |  |
|  |  |  |  |  |
| **Owns livestock, herds or farm animals** | No | 3366 (43.5) | 3354 (45.4) | 0.02 |
|  | Yes | 4366 (56.5) | 4039 (54.6) |  |
|  |  |  |  |  |
| **Source of drinking water** | Piped | 1772 (22.9) | 2387 (32.3) | <0.001 |
|  | Unpiped | 5960 (77.1) | 5006 (67.7) |  |
|  |  |  |  |  |
| **Household has a bed net** | No | 2253 (29.1) | 2299 (31.1) | 0.009 |
|  | Yes | 5479 (70.9) | 5094 (68.9) |  |
|  |  |  |  |  |
| **Slept under an LLIN last night** | No | 4957 (64.1) | 4677 (63.3) | 0.3 |
|  | Yes | 2775 (35.9) | 2716 (36.7) |  |
|  |  |  |  |  |
| **Insecticide of LLIN individual slept under last night** | Permethrin | 1691 (60.9) | 1641 (60.4) | 0.6 |
|  | Non-permethrin | 1084 (39.1) | 1074 (39.5) |  |
|  | Missing | 0 (0.0) | 1 (0.0) |  |
|  |  |  |  |  |
| **1 net per 1.8 household members** | No | 7705 (99.7) | 7357 (99.5) | 0.4 |
|  | Yes | 21 (0.3) | 29 (0.4) |  |
|  |  |  |  |  |
| **Anemia (women only)** | Not anemic | 2770 (66.5) | 2585 (65.9) | 0.6 |
|  | Mild | 1064 (25.6) | 1027 (26.2) |  |
|  | Moderate | 296 (7.1) | 283 (7.2) |  |
|  | Severe | 32 (0.8) | 23 (0.6) |  |
|  | Missing / NA | 1 (0.0) | 3 (0.1) |  |
|  |  |  |  |  |
| **Region** | Northern | 1025 (13.3) | 1896 (25.6) | <0.001 |
|  | Central | 3046 (39.4) | 2357 (31.9) |  |
|  | Southern | 3661 (47.3) | 3140 (42.5) |  |
|  |  |  |  |  |
| **Place of residence** | Urban | 1541 (19.9) | 1671 (22.6) | <0.001 |
|  | Rural | 6191 (80.1) | 5722 (77.4) |  |
|  |  |  |  |  |
| **Elevation (m)** | <500 | 1480 (19.1) | 1474 (19.9) | <0.001 |
|  | ≥ 500 & <1000 | 3190 (41.3) | 2828 (38.3) |  |
|  | ≥ 1000 & <1500 | 2891 (37.4) | 2881 (39.0) |  |
|  | ≥ 1500 | 171 (2.2) | 210 (2.8) |  |
|  |  |  |  |  |
| **Month of data collection** | October '15 | 1528 (19.8) | 1038 (14.0) | <0.001 |
|  | November '15 | 3070 (39.7) | 2005 (27.1) |  |
|  | December '15 | 1627 (21.0) | 904 (12.2) |  |
|  | January '16 | 1335 (17.3) | 3078 (41.6) |  |
|  | February '16 | 172 (2.2) | 368 (5.0) |  |
|  |  |  |  |  |
| **Proportion of cluster with bed nets** | mean (SD) | 0.68 (0.18) | 0.68 (0.19) | 0.5 |
|  |  |  |  |  |
| **Proportion of cluster that slept under an LLIN last night** | mean (SD) | 0.32 (0.16) | 0.33 (0.15) | <0.001 |
|  |  |  |  |  |
| * significance testing uses t-tests with an alpha=0.05 for continuous variables and chi-squared tests for categorical variables, comparing between strata of available and unavailable samples. | | | | |

**Supplementary Tables 5-8** found in Supplemental Excel file.

**Supplementary Table 9**. *P. falciparum* prevalence stratified by month and region.

| **Month** | **Region** | **N** | **Percent of samples collected during the month** | ***P. falciparum* prevalence** |
| --- | --- | --- | --- | --- |
| **January** | Northern | 278 | 9.0 | 0.291 |
|  | Central | 1094 | 35.5 | 0.282 |
|  | Southern | 1706 | 55.4 | 0.264 |
| **February** | Northern | 213 | 57.9 | 0.268 |
|  | Central | 137 | 37.2 | 0.467 |
|  | Southern | 18 | 4.9 | 0.444 |
| **October** | Northern | 65 | 6.3 | 0.246 |
|  | Central | 454 | 43.7 | 0.377 |
|  | Southern | 519 | 50.0 | 0.428 |
| **November** | Northern | 824 | 41.1 | 0.223 |
|  | Central | 486 | 24.2 | 0.358 |
|  | Southern | 695 | 34.7 | 0.273 |
| **December** | Northern | 516 | 57.1 | 0.188 |
|  | Central | 186 | 20.6 | 0.285 |
|  | Southern | 202 | 22.3 | 0.243 |


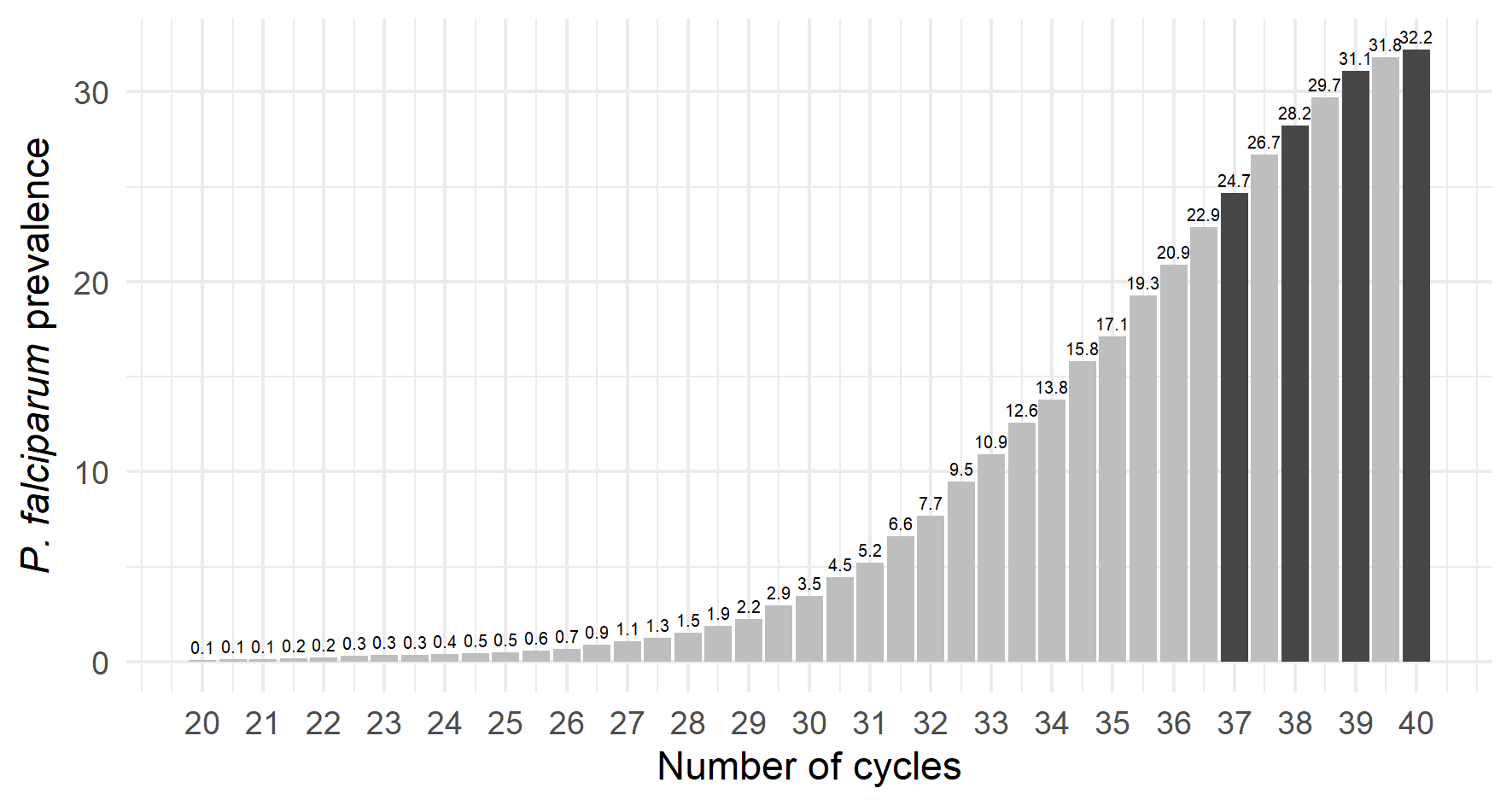


**Supplementary Figure 1**. Weighted *P. falciparum* prevalence at various C_T_ values (n=7,393). The q-PCR cycle number shows how many cycles occurred before the sample’s reaction curve crossed the threshold for a positive result.
